# Letter from the Editor in Chief

**DOI:** 10.19102/icrm.2019.100208

**Published:** 2019-02-15

**Authors:** Moussa Mansour


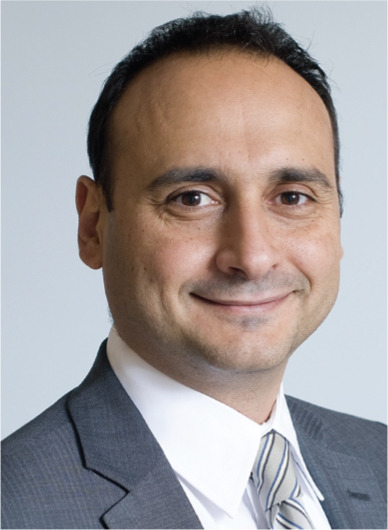


Dear Readers,

The recent science-packed AF Symposium held in Boston, MA was attended by more than 1,200 people from hospitals and health care industries in the United States and around the world and showcased novel atrial fibrillation (AF) ablation techniques and technologies by way of lectures, debates, live case presentations, and posters and abstracts. One of the highlights of the symposium this year was the late-breaking clinical trials. Of particular interest, two out of the four clinical trials presented covered novel energy sources for ablation.

Dr. Vivek Reddy of Mount Sinai Hospital in New York, NY presented outcomes of a first-in-human study on the use of an automated noncontact ultrasound imaging and ablation system for the treatment of AF. This novel tool uses low-intensity collimated ultrasound as an energy source. The robotic catheter moves under computer control and allows for both anatomical mapping of the left atrium and ablation. It relies on automated tissue thickness detection and other tissue characteristics to create continuous linear lesions without tissue contact. The catheter system is not yet approved by the United States Food and Drug Administration (FDA); the initial investigation was performed in the Czech Republic and enrolled 52 patients with drug-refractory, symptomatic paroxysmal AF. Manual radiofrequency ablation touch-up was required in a small number of patients. The primary safety endpoint of the study was met, with no occurrence of atrioesophageal fistula, pulmonary vein stenosis, cardiac perforation or tamponade, death, stroke, myocardial infarction, or thromboembolism. There was one instance of phrenic nerve injury. The effectiveness results were impressive at 12 months later, with 80% of the study subjects demonstrating chronic procedure success, defined as freedom from symptomatic atrial arrhythmia recurrence of more than 30 seconds, new class I or III antiarrhythmic drug use, and/or the performance of reablation after a three-month blanking period.

Separately, results of the Cryocure II study were presented by Dr. Tom De Potter of OLV Hospital in Aalst, Belgium. Similarly, the multielectrode catheter system used in this research is also not FDA-approved and the study was conducted in Europe. The catheter system uses near-critical nitrogen ultra-low-temperature technology to create deep and transmural lesions in a rapid fashion. Forty-eight patients with persistent and paroxysmal AF were enrolled. In addition to pulmonary vein isolation, patients with persistent AF underwent mitral isthmus ablation and cavotricupid isthmus ablation as needed. Freedom from AF was 100% in those with paroxysmal AF at 12 months and 94% in those with persistent AF at six months, respectively.

Importantly, though the research presented in the late-breaking clinical trials session was very impressive, like with any other new technology, the above preliminary results will need to be replicated in larger multicenter studies.

I hope that you find this issue of *The Journal of Innovations in Cardiac Rhythm Management* interesting.

Sincerely,


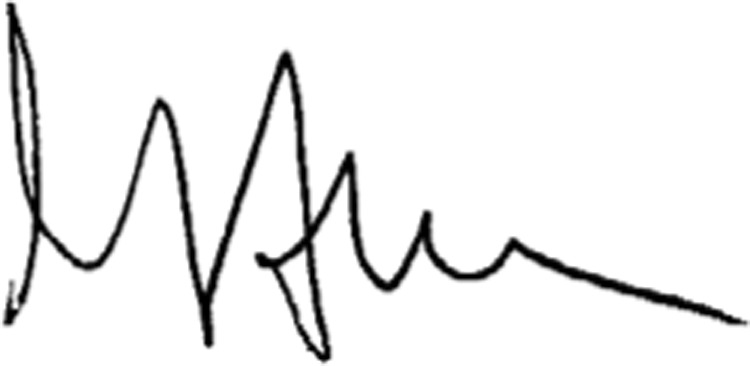


Moussa Mansour, md, fhrs, facc

Editor in Chief

The Journal of Innovations in Cardiac Rhythm Management

MMansour@InnovationsInCRM.com

Director, Atrial Fibrillation Program

Jeremy Ruskin and Dan Starks Endowed Chair in Cardiology

Massachusetts General Hospital

Boston, MA 02114

